# Effects of physical activity on fundamental motor skills and body composition in children and adolescents with intellectual and developmental disabilities: a systematic review and meta-analysis

**DOI:** 10.7717/peerj.20946

**Published:** 2026-04-01

**Authors:** Xiaohuan Tan, Lei Zhang, Dandan Wang, Xueping Wu

**Affiliations:** 1 Shanghai University of Sport, Shanghai, China; 2 University of Shanghai for Science and Technology, Shanghai, China

**Keywords:** Exercise, Children, Fundamental motor skills, Body composition, Meta-analysis, Disabilities

## Abstract

**Background:**

The development of fundamental motor skills (FMS) and changes in body composition among children and adolescents with intellectual and developmental disabilities (IDD) are an emerging public health issue. Physical activity serves as a dual protective mechanism, enhancing motor skills and optimizing body composition. Yet no systematic reviews have been published examining the effects of structured exercise programs on both FMS progression and weight management in this population.

**Methods:**

Web of Science, PubMed, Cochrane Library, and Medline databases were searched for peer-reviewed English-language literature, from the establishment of the database to October 1, 2025. Risk of bias was assessed using a modified Cochrane Collaboration’s tool and PEDro scale. The inclusion criteria comprised randomized or controlled trial that reported on FMS or body composition in children and adolescents (4–18 years) with IDD. Subgroup analyses were performed according to the type of physical activity.

**Results:**

Thirty-three studies (*n* = 5,245, average age ranging from 4 to 18 years) were included. Physical activity significantly improved FMS (standard mean difference (SMD) = 1.21, 95% CI [0.85–1.57], *p* < 0.001). Subgroup analyses showed significant improvements in locomotor skills (SMD = 1.13, 95% CI [0.83–1.42], *p* < 0.001) and object control skills (SMD = 0.87, 95% CI [0.57–1.17], *p* < 0.001). Among physical activity types, virtual reality (VR) games (SMD = 1.00, 95% CI [0.60–1.40], *p* < 0.001), motor skills (SMD = 1.79, 95% CI [1.11–2.47], *p* < 0.001), and sport-specific programs (SMD = 1.13, 95% CI [0.57–1.70], *p* < 0.001) improved the FMS. In terms of body composition, moderate intensity continuous training (MICT) significantly reduced body mass index (SMD = −0.29, 95% CI [−0.50 to −0.08], *p* = 0.006) and body fat percentage (SMD = −0.55, 95% CI [−1.01 to −0.09], *p* = 0.020). In contrast, the reduction in waist circumference was not significant (SMD = −0.32, 95% CI [−0.69 to 0.04], *p* = 0.080).

**Conclusion:**

Our findings identify interventions focused on motor skills and MICT as effective strategies for improving FMS and body composition in this population, which may lay a foundation for their long-term health. In contrast, VR games have limited empirical support based on our assessment of the evidence quality.

## Introduction

Fundamental motor skills (FMS) constitute the neurodevelopmental foundation for acquiring specialized movement competencies essential for physical activity and sports participation (Logan et al., 2012). Studies have demonstrated that FMS development does not emerge through natural developmental processes (Barnett et al., 2016), but requires structured learning experiences and systematic reinforcement (Logan et al., 2018). This developmental imperative holds particular significance given the established relationship between motor skills attainment and sustained physical activity engagement across the lifespan, a critical consideration in light of global health guidelines (Clark & Metcalf, 2002). The World Health Organization specifically advocates 60 min of daily moderate-to-vigorous physical activity (PA) for children and adolescents, explicitly including those with disabilities (World Health Organization, 2020), to mitigate chronic disease risks (*e.g.*, cardiometabolic disorders, oncological conditions, affective dysregulation) (Sutherland et al., 2021). However, epidemiological data have revealed that despite physical activity demonstrating dose-dependent reductions in chronic disease incidence (Rinehart, Jeste & Wilson, 2018), population-level physical activity participation remains suboptimal across pediatric subgroups, particularly those with neuromotor impairments (De, Small & Baur, 2008).

Intellectual and developmental disabilities (IDD) are neurodevelopmental disorders arising from central nervous system dysfunction (Patel et al., 2016), characterized by childhood-onset deficits in intellectual functioning and adaptive behaviors (Schalock, Luckasson & Tassé, 2021). Research indicates that neurological injury in the brain often causes varied degrees of motor dysfunction in children with IDD, such as delayed motor development and poorer motor coordination (Green et al., 2009). This neuromotor profile explains the pronounced FMS disparities observed in children with IDD, whose developmental lag is below that of neurotypical peers (Rintala & Loovis, 2013; Lloyd, 2016). Compounded by diminished physical activity motivation and elevated sedentary behaviors (Maïano, Hue & April, 2019), these movement deficiencies create a higher prevalence of obesity, which is a serious health issue (Maïano, 2011; Maïano et al., 2016; Wang et al., 2022). Lower levels of FMS are associated with lower physical activity levels and a higher risk of overweight/obesity (Barnett et al., 2016; Sutherland et al., 2021). Therefore, increasing sports engagement and reducing health issues may depend on improving FMS and body composition in children and adolescents with IDD.

The neuroplasticity-sensitive periods of early childhood and adolescence present critical windows for the development of FMS and prevention of obesity (Morgan et al., 2013). While some intervention studies report positive effects on motor skills (Bremer, Balogh & Lloyd, 2015; Ashori, Norouzi & Jalil-Abkenar, 2018), they often use small sample sizes or lack rigorous control, limiting the generalizability of their findings. Furthermore, these studies often focus narrowly on motor outcomes while neglecting concurrent physiological changes. Current research on FMS development in children and adolescents with IDD reveals significant gaps in evidence-based early intervention strategies (McGarty et al., 2018). While existing studies have established foundational knowledge regarding motor skill profiles in this population, the scientific community has predominantly focused on two distinct aspects: postural control investigations in pediatric populations and studies on adults with IDD (Maïano et al., 2014; Jacinto et al., 2023b; Jacinto et al., 2023a). First, systematic investigations into physical activity outcomes remain limited, particularly regarding body composition parameters such as body mass index (BMI) and waist circumference (WC) (Mirza et al., 2022). Second, studies directly comparing the efficacy of different physical activity modalities for optimizing these physiological indices are notably absent in the current literature (Oh, Son & Ha, 2025; Li & Hu, 2025). Furthermore, a recent systematic review and meta-analysis by Jacinto et al. (2023a) and Jacinto et al. (2023b) was limited to body composition outcomes (neglecting critical FMS) and conflated pediatric and adult data by including participants of any age (Jacinto et al., 2023b). Given that childhood and adolescence represent a critical neuroplastic window for FMS development, a synthesis that specifically isolates this younger population and integrates analyses of both motor and body composition outcomes is urgently needed.

## Methods

This review was performed in accordance with the Cochrane Handbook for Systematic Reviews of Interventions (version 6.1) and the 2020 checklist for the Preferred Reporting Items for Systematic Reviews and Meta-Analyses (PRISMA) guidelines (Page et al., 2021). Protocol registration occurred after duplicate topic verification in the PROSPERO database (ID: CRD42024509463), but before screening was completed and data extraction started.

### Eligibility criteria

The inclusion criteria were as follows: (1) a minimum four-week duration to ensure an adequate intervention dose, as evidence indicates this period is required to improve neuromuscular and body composition outcomes in youth (Behringer et al., 2010); (2) all participants had to be ≤18 years of age; (3) the measured outcomes had to include FMS (locomotor and object control skills) and body composition (BMI, body fat percentage, *etc.*); (4) the study design was a randomized or non-randomized controlled trial (RCT or CT) comparing physical activity against a control condition (*e.g.*, usual care, waitlist, or regular practice); (5) studies reported complete outcome data; and (6) studies were published in peer-reviewed journals. The exclusion criteria were as follows: (1) participants with uncontrolled chronic diseases, pathologies, or injuries; (2) unpublished literature, dissertations, conference abstracts, reviews, literature reviews, theses, and case reports; (3) studies with no full text available in English; and (4) the physical activity intervention was combined with other substantial components (*e.g.*, dietary, psychological, or lifestyle interventions) that could confound the isolated effect of exercise.

### Search strategy

The Web of Science, PubMed, Cochrane Library, and Medline databases were searched for studies published in English up to October 1, 2025 File S1. A ‘snowball search’ was conducted to identify additional articles. The reference lists of the relevant studies were also screened. To supplement the electronic search, a manual review of reference lists from pertinent publications and other reviews related to the topic (Maïano, Hue & April, 2019; Salse-Batán et al., 2022) was performed to locate further studies. Two reviewers (X T and L Z) independently assessed the identified publications for eligibility and any disagreements were resolved by a third reviewer (D W).

### Data extraction

Data extraction was independently conducted by two authors (X T and L Z), subsequently verified by a third researcher (D W). The review study criteria were based on the medical PICOS (Page et al., 2021) strategy comprising five main elements: (1) participant characteristics; (2) type of physical activity, volume, and intensity; (3) comparator (control group *vs.* experimental group); (4) outcome measurements, including pre- and post-test means and standard deviations for FMS and body composition, specifically noting the assessment tool used for FMS (*e.g.*, test of gross motor development and bruininks-oseretsky test); and (5) study design and key details such as the first author and country of the study. In cases where data were missing, the corresponding author of the study was contacted to request information. If the authors were unresponsive or unreachable, the study was excluded from analysis.

### Risk of bias and quality assessment

Risk of bias assessment was performed using a Cochrane Collaboration tool for assessing the risk of bias in randomized trials. Two authors (X T and L Z) independently assessed the included articles using the Physiotherapy Evidence Database (PEDro) scale, with any disagreements resolved by a third reviewer (D W). The PEDro scale consists of 11 items to assess the methodological quality of the studies (Maher et al., 2003). Studies with a total score of ≥ 6 were considered high quality, while those with a total score of < 5 were considered low quality (Maher et al., 2003).

### Statistical analysis

The meta package in R (version 4.3.0; R Core Team, 2023) was used to conduct the meta-analytic calculations aimed at quantifying the effect sizes of the experimental interventions (Harrer et al., 2021). These calculations were based on the precise extraction of means and standard deviations (SDs) from original research publications. The following formula was employed to calculate the change in SD (Lund, 1988):

*mean change* = *mean*_*post*_ –*mean*_*pre*_

$change~in~SD=\sqrt{S{D}_{pre}^{2}+S{D}_{post}^{2}-2r\times S{D}_{pre}\times S{D}_{post}}~$

where *SD*_*pre*_ is the pre-intervention *SD*, *SD*_*post*_ is the post-intervention *SD*, r is the pre-intervention and post-intervention correlation, and is within the range of participant correlations. The within-participant correlation was set at 0.5.

The pooled standardized mean difference (SMD) between groups was computed along with the 95% confidence interval (CI). Where applicable, subgroup analyses were performed based on several pre-specified moderators: the measurement tool used for FMS, the specific sub-dimensions of FMS, and the type of physical activity (categorized into fundamental motor skill interventions, sports-specific programs, exergaming/virtual reality (VR), and combined training, *etc.*). Sensitivity analyses were conducted by testing the significance of the results when removing one study at a time (using the leave-one-out method) to assess whether the findings were predominantly influenced by any single study. The *I*^2^ statistic was used to assess heterogeneity across studies, with *I*^2^ values > 25%, 50%, and 75% indicating low, moderate, and high heterogeneity, respectively. A random-effects model was applied when significant heterogeneity was present (*p* < 0.05, *I*^2^ > 50%); otherwise, a fixed-effects model was used (Higgins et al., 2003). Publication bias was assessed using Egger test (Duval & Tweedie, 2000). This study highlights the clinical relevance of predictive intervals, which enhance traditional meta-analysis by accounting for heterogeneity, thereby improving the practical application of research findings (Riley, Higgins & Deeks, 2011). The level of statistical significance was set at *p* < 0.05.

## Results

### Search results

**Figure 1:**
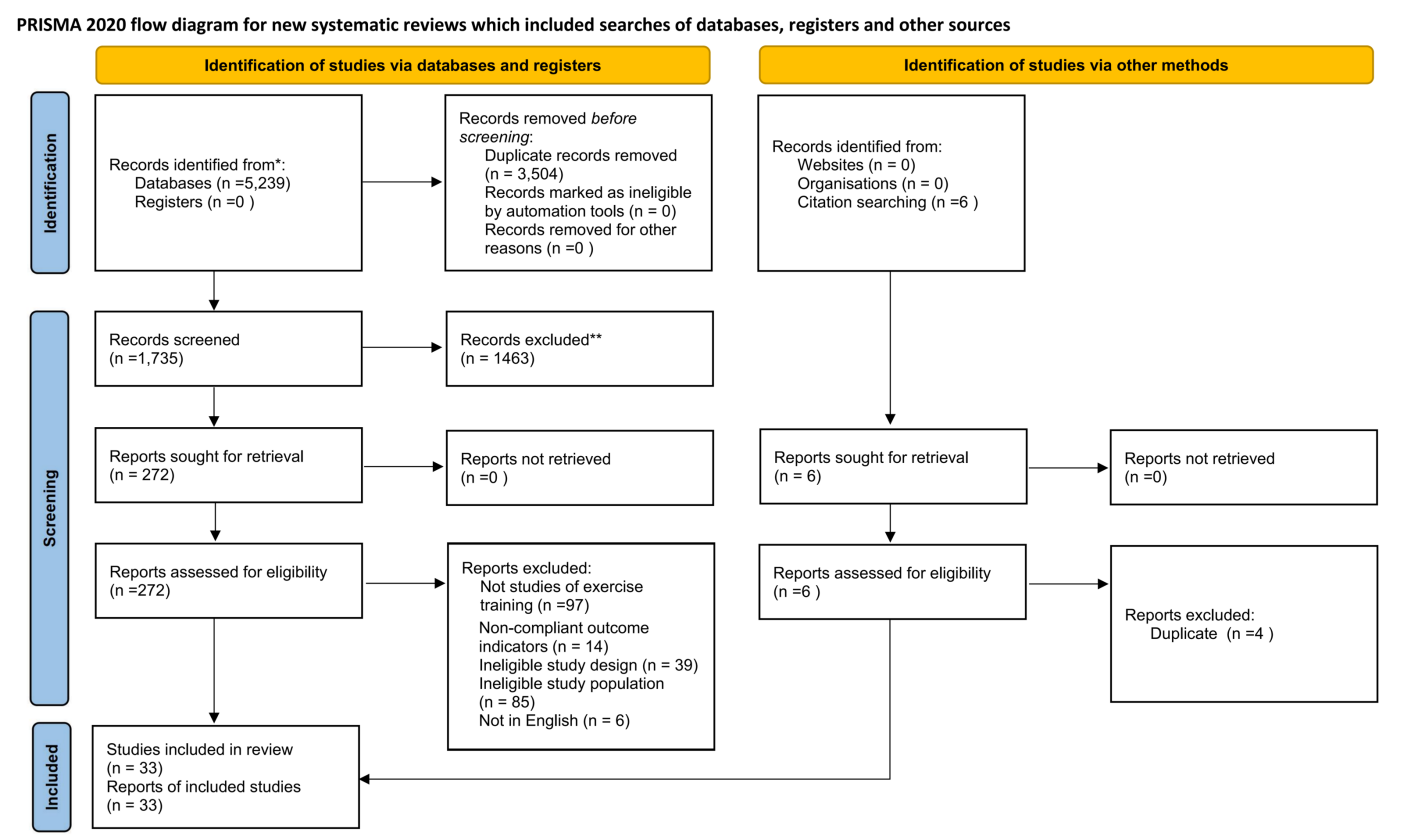
PRISMA flow chart of study selection.

A total of 5,245 papers were identified through searches. The databases searched included Web of Science (*n* = 2,223), PubMed (*n* = 1,676), Cochrane Library (*n* = 547), Medline (*n* = 793) and other methods (*n* = 6). A total of 272 reports were retrieved for full-text assessment. After a detailed review, 241 reports were excluded for the following reasons: not studies of exercise training (*n* = 97), ineligible study design (*n* = 39), ineligible study population (*n* = 85), non-compliant outcome indicators (*n* = 14), and not in English (*n* = 6). Consequently, 31 studies from the database search met the inclusion criteria. When combined with two additional studies identified through other sources, a total of 33 studies were finally included in this review (Fig. 1).

### Study characteristics

#### Participant characteristics

Overall, this study consisted of 1,278 children and adolescents with IDD, approximately half of whom were assigned to the experimental group and the other half to the control group. Group sample sizes ranged from five to 121 participants. Two studies (Pan et al., 2017; Zhang et al., 2021) exclusively reported male participants, while 15 studies (Elmahgoub et al., 2011; Lin & Wuang, 2012; Boer et al., 2014; Ashori, Norouzi & Jalil-Abkenar, 2018; Arabi et al., 2019; Xu et al., 2020; Naczk, Gajewska & Naczk, 2021; Yu et al., 2022; Pourmohamadreza-Tajrishi & Azadfallah, 2022; Kashi et al., 2023; Lin et al., 2023; Suarez-Villadat, Sadarangani & Villagra, 2023; Al-Nemr & Reffat, 2024; Mao et al., 2025; Wu et al., 2025) did not specify the gender of the participants. A total of 20 studies focused on children and adolescents with intellectual disabilities, 11 studies (Elmahgoub et al., 2011; Boer et al., 2014; Ashori, Norouzi & Jalil-Abkenar, 2018; Lau, Wang & Wang, 2020; Hsu et al., 2021; Zhang et al., 2021; Regaieg, Sahli & Kermarrec, 2021; Yu et al., 2022; Wang et al., 2022; Kashi et al., 2023; Lin et al., 2023; Karakaş, Eroğlu Kolayiş & Bayazıt, 2025) reported on children with an intelligence quotient below 70, eight studies (Lin & Wuang, 2012; Kong et al., 2019; Regaieg, Kermarrec & Sahli, 2020; Suarez-Villadat et al., 2020; Naczk, Gajewska & Naczk, 2021; Raghupathy, Divya & Karthikbabu, 2022; Suarez-Villadat, Sadarangani & Villagra, 2023; Al-Nemr & Reffat, 2024) involved participants with Down syndrome, and one study (Rubin et al., 2019) focused on children with Prader-Willi syndrome. Additionally, 11 studies (Bremer, Balogh & Lloyd, 2015; Pan et al., 2017; Arabi et al., 2019; Hassani et al., 2020; Greco, 2020; Marzouki et al., 2022; Pourmohamadreza-Tajrishi & Azadfallah, 2022; Kaplánová et al., 2022; Castaño et al., 2024; Mao et al., 2025; Wu et al., 2025) included children with autism, and one study (Xu et al., 2020) involved both children with Down syndrome and those with ASD. Of the 33 studies included in this review, only one study (Raghupathy, Divya & Karthikbabu, 2022) reported the use of intention-to-treat analyses (Table 1).

**Table 1:** Basic characteristics of the included studies.

Author	Country	Design	Age, y	Sample		Diagnosis (IQ)	Training regimen	Dose; duration; frequency	Measures
				EG (M:F)	CG (M:F)		Type		
Al-Nemr & Reffat (2024)	Egypt	RCT	8–10	20 (NA)	20 (NA)	DS	Pilates exercises	12 wk, 3 session, 45 min	BOT
Arabi et al. (2019)	Iran	RCT	6–12	15 (NA)	15 (NA)	ASD	Motor Skills	10 wk, 3 sessions, 50 min	TGMD
Ashori, Norouzi & Jalil-Abkenar (2018)	Iran	CT	8–10	13 (NA)	13 (NA)	ID (61.35)	Motor Skills	16 wk, 3 sessions, 45 min	BOT
Boer et al. (2014)	Belgium	RCT	17 ± 3.0	17 (NA)	14 (NA)	ID(59 ± 8.6)	Sprint Interval Training	15 wk, 2 sessions, 40 min	BMI, BF%
Bremer, Balogh & Lloyd (2015)	Canada	CT	4–6	5 (5:0)	3 (2:1)	ASD	Motor Skills	12 wk, 1 session, 60 min	PDMS-2
Castaño et al. (2024)	Colombia	RCT	4–7	10 (8:2)	10 (9:1)	ASD	Structured Motor Skills	8 wk, 3 sessions, 60 min	FMS:ADC
Elmahgoub et al. (2011)	Belgium	CT	14–22	15 (NA)	15 (NA)	ID (45–70)	Strength and Endurance	10 wk, 3 sessions, 50 min	BMI, WC
Greco (2020)	Italy	RCT	8–11	12 (10:2)	12 (10:2)	ASD	Motor Skills	12 wk, 2 sessions, 70 min	BOT
Hassani et al. (2020)	Iran	RCT	8–11	10 (6:4)	9 (6:3)	ASD	Motor Skills	8 wk, 2 sessions, 60 min	BOT
Hsu et al. (2021)	China	RCT	15–17	27 (23:4)	27 (23:4)	ID (50–70)	Floor Hockey	12 wk, 3 sessions, 90 min	BOT
Kaplánová et al. (2022)	Slovakia	CT	5–10	10 (9:1)	10 (8:2)	ASD	Motor Skills	8 wk, 2 sessions, 30 min	TGMD
Karakaş, Eroğlu Kolayiş & Bayazıt (2025)	Turkiye	RCT	8–11	10 (3:7)	11 (6:5)	ID	Hybrid Sports	24 wk, 1 session, 60 min	BOT
Kashi et al. (2023)	Iran	RCT	7–18	18 (NA)	18 (NA)	LID	Hybrid Sports	12 wk, 3 sessions, 70 min	BOT, BMI
Kong et al. (2019)	China	RCT	10–18	17 (15:2)	19 (16:3)	DS	TC and AT (dance)	12 wk, 2 sessions, 60 min	BMI
Lau, Wang & Wang (2020)	China	RCT	8–18	121 (NA)	73 (NA)	ID (55–70)	VR Games	12 wk, 2 sessions, 30 min	BOT, BMI, BF%
Lin & Wuang (2012)	China	RCT	13–18	46 (NA)	46 (NA)	DS	VR Games	6 wk, 3 sessions, 30 min	BMI
Lin et al. (2023)	China	RCT	15–18	17 (NA)	17 (NA)	MID	Rope Skipping	8 wk, 3 sessions, 50 min	BMI
Mao et al. (2025)	China	RCT	7.60 ± 2.81	15 (NA)	14 (NA)	ASD	Ball Sport	12 wk, 5 sessions, 45 min	BMI
Pourmohamadreza-Tajrishi & Azadfallah (2022)	Iran	RCT	8–10	15 (NA)	15 (NA)	ASD	Motor Skills	8 wk, 2 sessions, 45 min	TGMD-2
Marzouki et al. (2022)	Africa	RCT	6.3 ± 0.5	10 (8:2)	8 (6:2)	ASD	Aquatic Training	8 wk, 2 sessions, 50 min	TGMD-2, BMI
Naczk, Gajewska & Naczk (2021)	Poland	RCT	14.9 ± 2.3	11 (NA)	11 (NA)	DS	Swimming Training	33 wk, 3 sessions, 70–90 min	BMI, BF%
Pan et al. (2017)	China	RCT	6–12	11 (11:0)	11 (11:0)	ASD	Motor Skills	12 wk, 2 sessions, 70 min	BOT
Raghupathy, Divya & Karthikbabu (2022)	Indian	RCT	6–10	18 (11:7)	18 (10:8)	DS	Indian Dance	6 wk, 3 sessions, 60 min	TGMD
Regaieg, Kermarrec & Sahli (2020)	France	CT	8.8 ± 1.0	13 (10:3)	15 (10:5)	DS (55–70)	VR Games	10 wk, 3 sessions, 60 min	TGMD
Regaieg, Sahli & Kermarrec (2021)	France	CT	7–10	12 (7:5)	12 (6:6)	ID (50–70)	VR Games	10 wk, 3 sessions, 60 min	TGMD
Rubin et al. (2019)	USA	RCT	8–16	34 (22:12)	11 (3:8)	PWS	VR Games	24 wk, 4 sessions, 25–45 min	BMI, BF%
Suarez-Villadat et al. (2020)	Spain	RCT	12–15	15 (8:7)	30 (17:13)	DS	Swimming Training	36 wk, 3 sessions, 50 min	BMI, BF%, WC
Suarez-Villadat, Sadarangani & Villagra (2023)	Spain	RCT	14.2 ± 2.0	24 (NA)	25 (NA)	DS	VR Games	20 wk, 3 sessions, 60 min	BMI, WC, BF%
Wu et al. (2025)	China	RCT	6–12	20 (NA)	20 (NA)	ASD	VR Games	12 wk, 3 sessions, 45 min	TGMD
Wang et al. (2022)	China	RCT	12-18	15 (9:6)	15 (13:2)	ID	AT and RT	12 wk, 2 sessions, 60 min	BMI, BF%
Xu et al. (2020)	China	CT	7.2	12 (NA)	10 (NA)	ASD,DS	Rhythmic Gymnastics	16 wk, 3 sessions, 50 min	BMI
Yu et al. (2022)	China	RCT	12–18	41 (NA)	40 (NA)	ID (35–69)	AT and RT	36 wk, 2 sessions, 45 min	BMI, BF%, WC
Zhang, Wang & Wu (2022)	China	RCT	7 -12	24 (24:0)	18 (18:0)	ID(20-35)	Floor Hockey	48 wk, 5 sessions, 60 min	TGMD

**Notes.**

Abbreviations ASD autism spectrum disorder AT and RT Aerobic training and resistance training VR Game Virtual Reality Games BMI body mass index BOT Bruininks-Oseretsky test of motor proficiency CG control group CT controlled trial DS Down syndrome EG experimental group BF% body fat percentage FMS fundamental motor skills ID intellectual disability IQ Intelligent Quotient LID Light intellectual disability M:F male: female MID Moderate intellectual disability PWS Prader-willi syndrome RCT randomized controlled trial TGMD test of gross motor development WC waist circumference NA not applicable

#### Interventions and procedures

A total of nine studies (Bremer, Balogh & Lloyd, 2015; Pan et al., 2017; Ashori, Norouzi & Jalil-Abkenar, 2018; Arabi et al., 2019; Hassani et al., 2020; Greco, 2020; Pourmohamadreza-Tajrishi & Azadfallah, 2022; Kaplánová et al., 2022; Castaño et al., 2024) included interventions focused on motor skills acquisition, 12 studies (Kong et al., 2019; Suarez-Villadat et al., 2020; Xu et al., 2020; Hsu et al., 2021; Naczk, Gajewska & Naczk, 2021; Zhang, Wang & Wu, 2022; Raghupathy, Divya & Karthikbabu, 2022; Marzouki et al., 2022; Lin et al., 2023; Al-Nemr & Reffat, 2024; Karakaş, Eroğlu Kolayiş & Bayazıt, 2025; Mao et al., 2025) included sports-specific programs (including three swimming programs, two floor hockey, one aerobic dance, one rope skipping exercise, one Tai Chi exercise, one artistic gymnastics, one Pilates exercises and two ball sport), seven studies (Lin & Wuang, 2012; Rubin et al., 2019; Regaieg, Kermarrec & Sahli, 2020; Lau, Wang & Wang, 2020; Regaieg, Sahli & Kermarrec, 2021; Suarez-Villadat, Sadarangani & Villagra, 2023; Wu et al., 2025) included interventions involving games, four studies (Elmahgoub et al., 2011; Yu et al., 2022; Wang et al., 2022; Kashi et al., 2023) reported on combined physical activity (including two combining aerobic and resistance training, one combining resistance and endurance training, and one combining aerobic, balance, and sensory perception exercises), and one study (Boer et al., 2014) included sprinting interval interventions. The duration of the interventions ranged from 6 to 48 weeks, with 8–12 weeks being the most common. Training frequency varied between one to five times per week, with 15 studies conducting training three times per week. The treatment duration ranged from 25 to 240 min. Twenty studies (Bremer, Balogh & Lloyd, 2015; Pan et al., 2017; Ashori, Norouzi & Jalil-Abkenar, 2018; Rubin et al., 2019; Arabi et al., 2019; Hassani et al., 2020; Greco, 2020; Regaieg, Kermarrec & Sahli, 2020; Xu et al., 2020; Hsu et al., 2021; Zhang et al., 2021; Naczk, Gajewska & Naczk, 2021; Regaieg, Sahli & Kermarrec, 2021; Raghupathy, Divya & Karthikbabu, 2022; Pourmohamadreza-Tajrishi & Azadfallah, 2022; Kaplánová et al., 2022; Suarez-Villadat, Sadarangani & Villagra, 2023; Al-Nemr & Reffat, 2024; Karakaş, Eroğlu Kolayiş & Bayazıt, 2025; Wu et al., 2025) did not report how exercise intensity was monitored, whereas nine studies (Elmahgoub et al., 2011; Lin & Wuang, 2012; Boer et al., 2014; Kong et al., 2019; Lau, Wang & Wang, 2020; Yu et al., 2022; Wang et al., 2022; Kashi et al., 2023; Lin et al., 2023) provided detailed information on exercise intensity monitoring. Of these, nine studies (Elmahgoub et al., 2011; Lin & Wuang, 2012; Boer et al., 2014; Kong et al., 2019; Suarez-Villadat et al., 2020; Yu et al., 2022; Wang et al., 2022; Kashi et al., 2023; Mao et al., 2025) used heart rate to monitor intensity, primarily targeting 60% to 85% of maximum heart rate (American College of Sports Medicine (ACSM), 2024), one study (Lin et al., 2023) used the Borg Rating of Perceived Exertion (RPE) scale, and three studies (Lau, Wang & Wang, 2020; Marzouki et al., 2022; Castaño et al., 2024) considered exercise intensity in their assessments. The intervention progression in these studies involved an increase in exercise intensity (Table 1).

#### Risk of bias assessment

**Figure 2:**
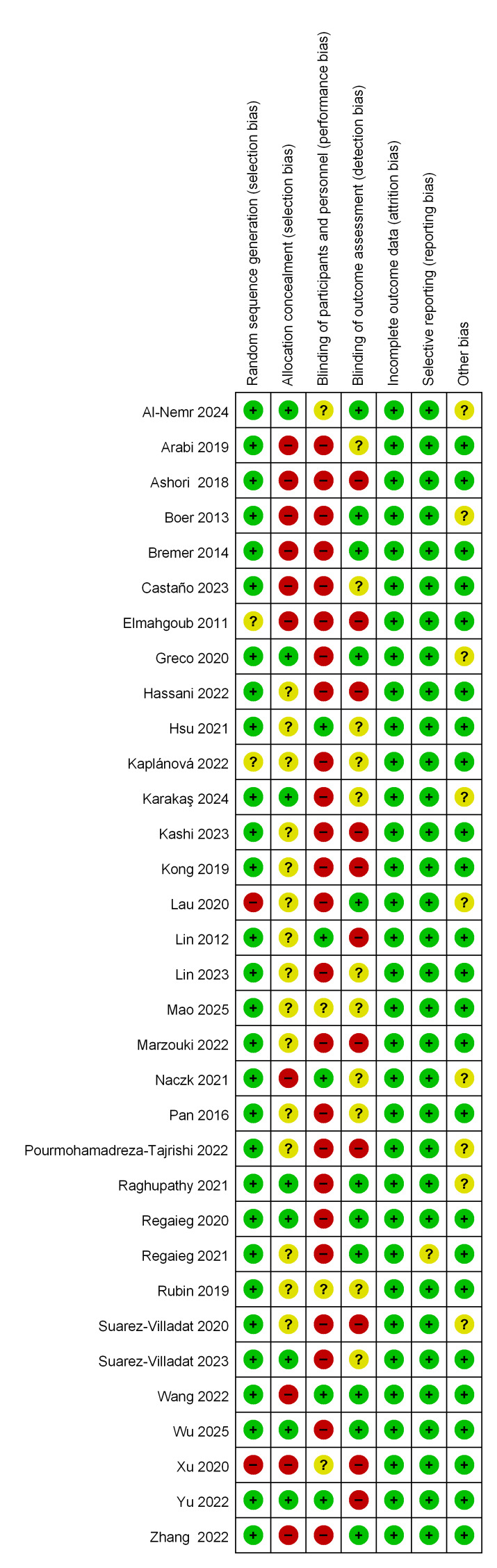
Summary of risk bias.

Two reviewers independently assessed the studies using the Cochrane Risk of Bias Assessment Tool. As shown in Fig. 2, the included studies were generally considered to have a low risk of bias. In studies involving special populations, achieving blinding of both participants and practitioners to the intervention is challenging. Two studies (Hsu et al., 2021; Wang et al., 2022) blinded participants to the intervention, two studies (Lin & Wuang, 2012; Yu et al., 2022) blinded the intervention trainers, and ten studies (Boer et al., 2014; Greco, 2020; Regaieg, Kermarrec & Sahli, 2020; Lau, Wang & Wang, 2020; Zhang et al., 2021; Regaieg, Sahli & Kermarrec, 2021; Yu et al., 2022; Raghupathy, Divya & Karthikbabu, 2022; Wang et al., 2022; Al-Nemr & Reffat, 2024) blinded evaluators.

#### Quality of included studies

Of the 33 studies included in the review, 13 studies (Lin & Wuang, 2012; Boer et al., 2014; Greco, 2020; Regaieg, Kermarrec & Sahli, 2020; Hsu et al., 2021; Regaieg, Sahli & Kermarrec, 2021; Yu et al., 2022; Zhang, Wang & Wu, 2022; Raghupathy, Divya & Karthikbabu, 2022; Wang et al., 2022; Suarez-Villadat, Sadarangani & Villagra, 2023; Al-Nemr & Reffat, 2024; Wu et al., 2025) had a total score of ≥ 6, 13 studies (Bremer, Balogh & Lloyd, 2015; Pan et al., 2017; Ashori, Norouzi & Jalil-Abkenar, 2018; Kong et al., 2019; Arabi et al., 2019; Hassani et al., 2020; Naczk, Gajewska & Naczk, 2021; Marzouki et al., 2022; Pourmohamadreza-Tajrishi & Azadfallah, 2022; Castaño et al., 2024; Kashi et al., 2023; Lin et al., 2023; Karakaş, Eroğlu Kolayiş & Bayazıt, 2025) had a total score of five, and seven studies (Elmahgoub et al., 2011; Rubin et al., 2019; Suarez-Villadat et al., 2020; Xu et al., 2020; Lau, Wang & Wang, 2020; Kaplánová et al., 2022; Mao et al., 2025) had a total score of ≤ 4. Overall, studies assessing special populations were of moderately high quality (Table 2). Assessments were completed independently by two reviewers (X T and L Z) while any observed differences were resolved *via* discussion and agreement.

**Table 2:** Physiotherapy Evidence Database (PEDro) score of the included studies.

Study	Eligibility criteria	Random allocation	Concealed allocation	Similar baseline	Blinded subjects	Blinded therapists	Blinded assessors	Dropout <15%	Intention to treat	Between group statistics	Point measures	Total Score Items
Al-Nemr & Reffat (2024)	1	1	1	1	0	0	1	1	0	1	1	7
Arabi et al. (2019)	1	1	0	1	0	0	0	1	0	1	1	5
Ashori, Norouzi & Jalil-Abkenar (2018)	1	1	0	1	0	0	0	1	0	1	1	5
Boer et al. (2014)	1	1	0	1	0	0	1	1	0	1	1	6
Bremer, Balogh & Lloyd (2015)	1	1	0	1	0	0	0	1	0	1	1	5
Castaño et al. (2024)	1	1	0	1	0	0	0	1	0	1	1	5
Elmahgoub et al. (2011)	1	0	0	1	0	0	0	1	0	1	1	4
Greco (2020)	1	1	1	1	0	0	1	1	0	1	1	7
Hassani et al. (2020)	1	1	0	1	0	0	0	1	0	1	1	5
Hsu et al. (2021)	1	1	0	1	1	0	0	1	0	1	1	6
Kaplánová et al. (2022)	1	0	0	1	0	0	0	1	0	1	1	4
Karakaş, Eroğlu Kolayiş & Bayazıt (2025)	1	1	0	1	0	0	0	1	0	1	1	5
Kashi et al. (2023)	1	1	0	1	0	0	0	1	0	1	1	5
Kong et al. (2019)	1	1	0	1	0	0	0	1	0	1	1	5
Lau, Wang & Wang (2020)	1	0	0	0	0	0	1	1	0	1	1	4
Lin & Wuang (2012)	1	1	0	1	0	1	0	1	0	1	1	6
Lin et al. (2023)	1	1	0	1	0	0	0	1	0	1	1	5
Mao et al. (2025)	1	1	0	1	0	0	0	0	0	1	1	4
Pourmohamadreza-Tajrishi & Azadfallah (2022)	1	1	0	1	0	0	0	1	0	1	1	5
Marzouki et al. (2022)	1	1	0	1	0	0	0	1	0	1	1	5
Naczk, Gajewska & Naczk (2021)	1	1	0	1	0	0	0	1	0	1	1	5
Pan et al. (2017)	1	1	0	1	0	0	0	1	0	1	1	5
Raghupathy, Divya & Karthikbabu (2022)	1	1	1	1	0	0	1	1	1	1	1	8
Regaieg, Kermarrec & Sahli (2020)	1	1	1	1	0	0	1	0	0	1	1	6
Regaieg, Sahli & Kermarrec (2021)	1	1	0	1	0	0	1	1	0	1	1	6
Rubin et al. (2019)	1	1	0	1	0	0	0	0	0	1	1	4
Suarez-Villadat et al. (2020)	1	1	0	1	0	0	0	0	0	1	1	4
Suarez-Villadat, Sadarangani & Villagra (2023)	1	1	1	1	0	0	0	1	0	1	1	6
Wu et al. (2025)	1	1	1	1	0	0	1	1	0	1	1	7
Wang et al. (2022)	1	1	0	1	1	0	1	1	0	1	1	6
Xu et al. (2020)	1	0	0	1	0	0	0	1	0	1	1	4
Yu et al. (2022)	1	1	1	1	0	1	1	0	0	1	1	7
Zhang, Wang & Wu (2022)	1	1	0	1	0	0	1	1	0	1	1	6

### Meta-Analysis

#### Primary outcomes

**Figure 3:**
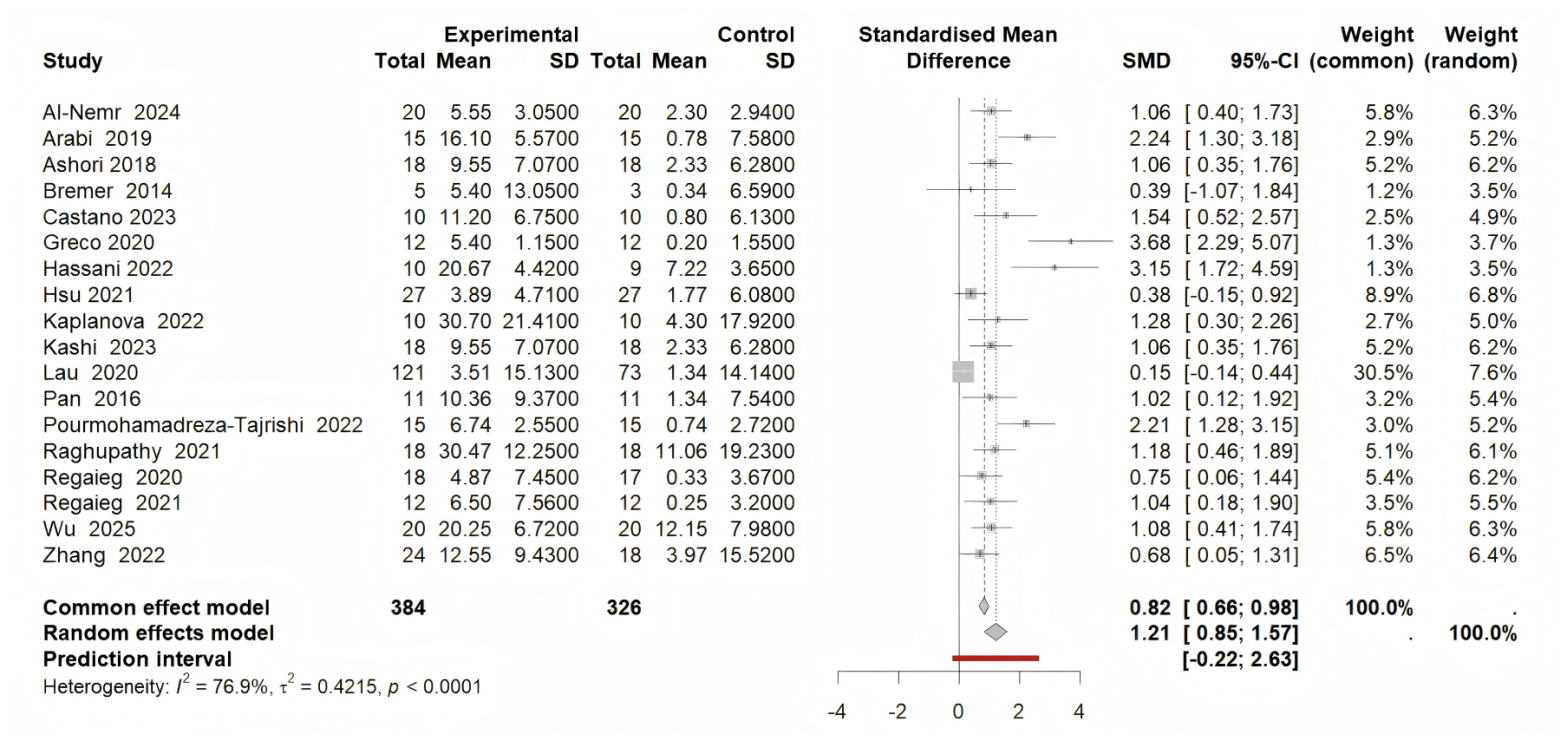
Forest plot for the effect of physical activity on FMS.

Our meta-analysis indicated that the combined effect of 18 interventions with a total of 710 participants significantly improved fundamental motor skills (SMD = 1.21, 95% CI [0.85–1.57], *p* < 0.001, *I*^2^ = 76%). There was a high heterogeneity among the studies (Fig. 3). Sensitivity analyses showed a combined effect size of SMD = 1.28, (95% CI [0.94–1.62], *p* < 0.001, *I*^2^ = 63%) when studies were excluded one by one, with the exclusion of Lau et al. accounting for this change.

#### Subgroup analysis

##### Subclasses of motor skills.

**Figure 4:**
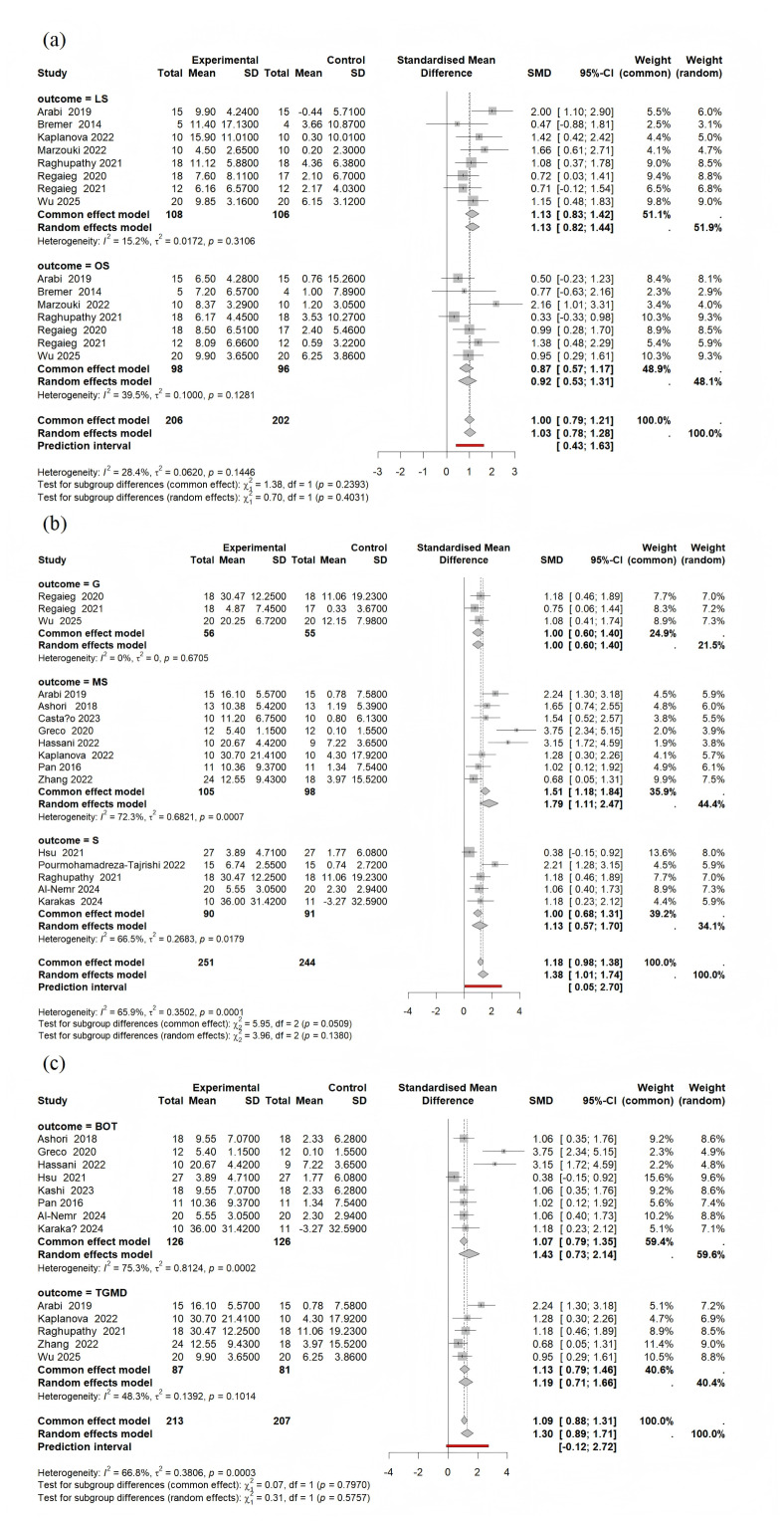
Forest plot of subgroup analyses for (A) FMS subclasses, (B) physical activity type, and (C) FMS measurement tools. Note: LS, locomotor skills; OS, object control skills; G, VR games; MS: motor skills; S, sports -specific programs; BOT, bruininks- oseretsky test of motor proficiency; TGMD, test of gross motor development.

The combined effect sizes of the eight studies, which included 214 participants, were significant for locomotor skills (SMD = 1.13, 95% CI [0.83–1.42], *p* < 0.001, *I*^2^ = 15%). For object control skills, the combined effect sizes were significant across the seven studies, with a total of 194 participants (SMD = 0.87, 95% CI [0.57–1.17], *p* < 0.001, *I*^2^ = 39%) (Fig. 4A).

##### Type of intervention.

Subgroup analysis of the effect on motor skills, based on the physical activity subcategory, revealed several significant findings. Specifically, virtual reality (VR) games significantly improved motor skills (SMD = 1.00, 95% CI [0.60–1.40], *p* < 0.001, *I*^2^ = 0%). Interventions directly targeting motor skills also demonstrated a significant positive effect on motor skills (SMD = 1.79, 95% CI [1.11–2.47], *p* < 0.001, *I*^2^ = 72%). Sports-specific programs significantly improved motor skills (SMD = 1.13, 95% CI [0.57–1.70], *p* < 0.001, *I*^2^ = 66%) (Fig. 4B).

##### Measurement tools.

Subgroup analyses were conducted based on FMS measurement tools. The bruininks-oseretsky test (BOT) of motor proficiency assesses, including gross motor, fine motor, and motor accuracy, while the test of gross motor development (TGMD) specifically focuses on gross motor skills. For the eight studies that used BOT, the combined effect size was significant (SMD = 1.43, 95% CI [0.73–2.14], *p* < 0.001, *I*^2^ = 75%). For the five studies that used TGMD, the combined effect size was also significant (SMD = 1.13, 95% CI [0.79–1.46], *p* < 0.001, *I*^2^ = 48%), suggesting that the interventions produced a significant effect on motor skills (Fig. 4C).

#### Secondary outcomes

**Figure 5:**
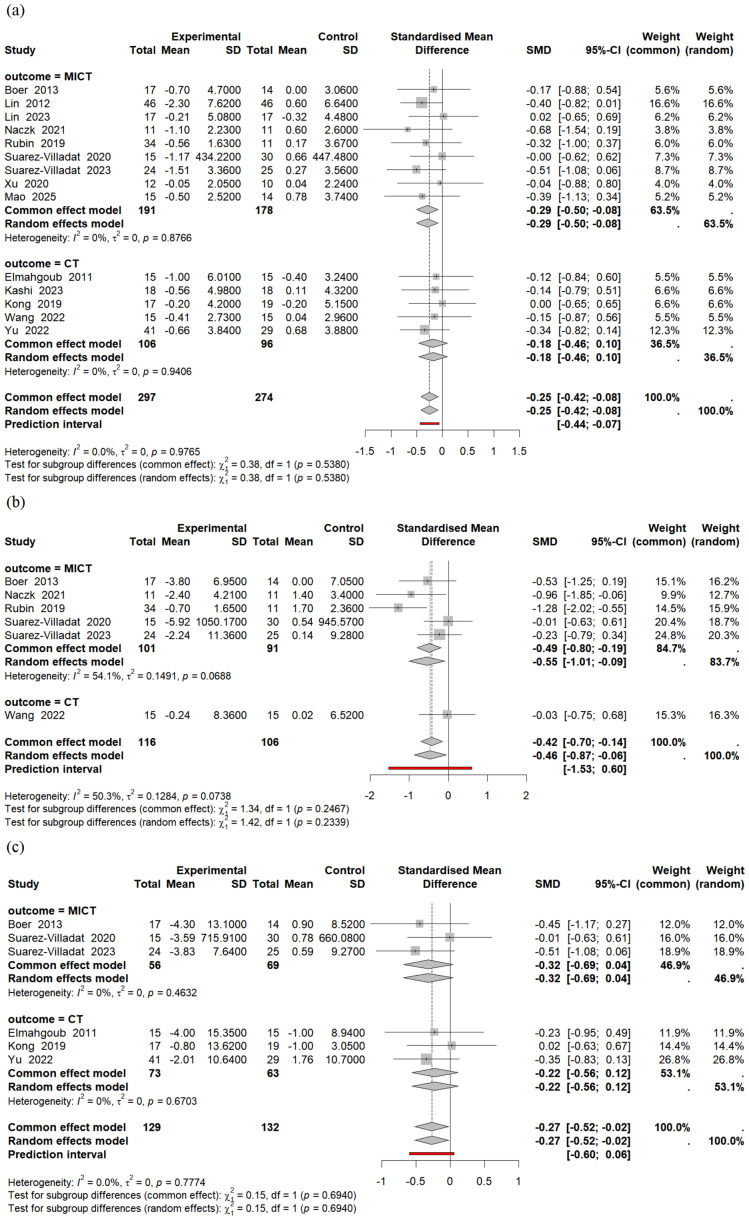
Forest plots of the effect of physical activity on (A) body mass index (BMI), (B) body fat percentage, and (C) waist circumference (WC).

Our meta-analysis found that MICT had a significant effect on BMI, with a combined effect size of SMD = −0.29 (95% CI [−0.50 to −0.08], *p* = 0.006, *I*^2^ = 0%) (Fig. 5A). MICT also significantly reduced body fat percentage (SMD = −0.55, 95% CI [−1.01 to −0.09], *p* = 0.020, *I*^2^ = 54%) (Fig. 5B). However, MICT did not significantly reduce waist circumference (WC) (SMD = −0.32, 95% CI [−0.69 to 0.04], *p* = 0.080, *I*^2^ = 0%) (Fig. 5C). Furthermore, combined training did not show significant improvements in body composition.

**Figure 6:**
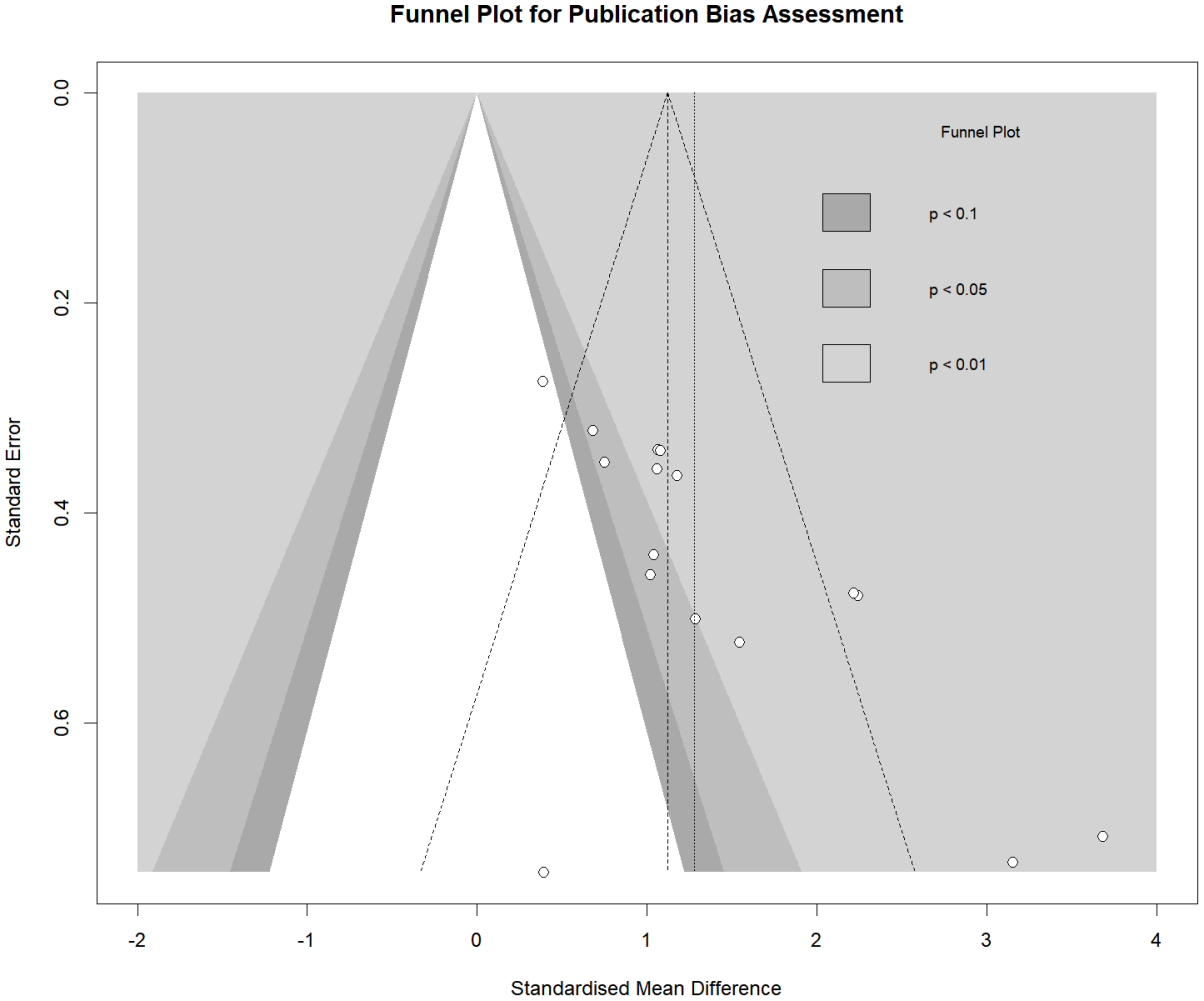
Funnel plot of FMS.

#### Publication bias

The results remained consistent across various sensitivity analyses. The Egger’s test indicated no publication bias for locomotor skills (*p* = 0.202), object control skills (*p* = 0.645), body fat percentage (*p* = 0.191), WC (*p* = 0.583), VR games (*p* = 0.841), the Test of Gross Motor Development (TGMD) (*p* = 0.103), and BMI (*p* = 0.253). However, the Egger’s test suggested some evidence of publication bias for FMS (*p* = 0.001) (Fig. 6), BOT (*p* = 0.001) and motor skills interventions (*p* = 0.003).

## Discussion

The present systematic review and meta-analysis revealed significant efficacy of exercise programs in enhancing fundamental movement skills (FMS) and improving body composition among children and adolescents with intellectual and developmental disabilities (IDD). These results further reinforce the positive influence of physical activity on motor skills in individuals with intellectual and developmental disabilities, which is consistent with prior research (Malekpour et al., 2012; Maïano, Hue & April, 2019; Özkan & Kale, 2021). The studies included in this review reported intervention durations ranging from 6 to 48 weeks, with two to three sessions per week, each lasting between 30 and 60 min. These findings highlight the importance of systematically teaching each individual skill component and providing individuals with IDD ample opportunities to repeat movements, thereby facilitating skill mastery (Kavanagh et al., 2023).

However, the studies included in this meta-analysis exhibited substantial heterogeneity. Further investigation of the sources of heterogeneity revealed several potential factors. First, a primary source is the significant methodological variability, particularly in the measurement tools used to assess the primary outcomes. For example, the included studies employed different instruments. TGMD primarily assesses gross motor skills (Nagy et al., 2023), whereas BOT evaluates both fine and gross motor skills. Combining effect sizes derived from instruments that may capture different facets or definitions of motor proficiency constitutes a form of ‘conceptual heterogeneity’. This not only directly contributes to the high statistical heterogeneity observed, but more importantly, it complicates the interpretation of the pooled effect size. Second, the inadequate sample size in some studies limits the generalizability of the findings and impedes direct comparison with the outcomes of the experimental group. Additionally, the heterogeneity may, in part, be attributed to variations in the severity of disability among participants. Given these differences in characteristics, it is not surprising that variability exists in the support needs within this population (Soenen, Van Berckelaer-Onnes & Scholte, 2016). Newell’s constraints model of motor development (Gkotzia, Venetsanou & Kambas, 2017) suggests that proficiency in FMS tends to decrease as the severity of disability increases in children and adolescents with intellectual and developmental disabilities (Newell, 1991). This highlights a significant gap in the current research that warrants further investigation.

Our results demonstrate that both types of skill interventions improve various outcomes, but the higher heterogeneity observed in object control skills suggests that variability between studies may influence the stability of these effects (Zhang et al., 2021). Empirical evidence from several studies indicates a positive correlation between higher object control skills and a greater propensity for children to engage in more structured movement patterns (Barnett et al., 2011; Westendorp et al., 2011). Locomotor tasks such as walking, running, and hopping are typically characterized by stereotypical movements (Goodway & Branta, 2003). In contrast, mastery of object control skills is recognized as a more complex process, often categorized as ‘open skills’. These skills are significantly influenced by environmental factors including the presence of external objects and interactions with other individuals. The execution of such skills requires adaptability to changing environmental conditions and engagement of higher-order cognitive processes (Houwen et al., 2007). Furthermore, divergent training approaches likely contributed to the observed heterogeneity. Structured, instructor-led programs prioritize biomechanical precision through repetitive task practice in closed environments. Conversely, game-based approaches emphasize adaptability to dynamic constraints, which is a core requirement for object control as an ‘open skill.’ This pedagogical diversity suggests that while structured repetitive practice improved technical execution in the included studies, game-based training may more effectively engage the higher-order cognitive processes essential for real-world mastery.

Our subgroup analysis found that various types of physical activity can significantly improve FMS in children and adolescents with IDD. Regarding the types of physical activity, 18 studies focused on FMS, two studies employed VR games, and three studies emphasized interventions targeting specific sports. Compared with VR games, interventions targeting motor skills and specific sports were found to be more effective in a study of 80 children with intellectual disabilities (Malekpour et al., 2012). This finding contrasts with a previous study involving 20 children with intellectual disabilities (Regaieg, Kermarrec & Sahli, 2020), which reported that structured and supervised game programs designed specifically to enhance object control skills may lead to substantial improvements in motor skills. Despite this inconsistency, the proposed mechanism for this enhancement suggests that, due to intellectual limitations, individuals in this population primarily engage passively in activities. To effectively consolidate and master these movements, structured physical activity designs are essential (Kesumawati et al., 2020), along with simple guidance and repeated practice (Arslan, Ince & Akyüz, 2020). These findings underscore the critical need to integrate structured and engaging exercise programs into routine care and therapeutic strategies for children with intellectual and developmental disabilities (Kaplánová et al., 2022).

Our secondary outcomes demonstrated that physical activity can significantly reduce BMI and body fat percentage in children with IDD. However, it is important to interpret the BMI finding with caution. BMI is not the optimal method for assessing body composition changes in response to physical activity, as it cannot differentiate between fat mass and lean muscle mass. Therefore, the significant reduction observed in body fat percentage provides a more precise and appropriate evaluation of the positive effects of these interventions. The findings of the current study contradict a systematic review (Harris et al., 2015), which reported no significant reduction in BMI following physical activity among children and adolescents with intellectual disabilities. Further analysis of the underlying causes revealed that the study examined physical activity durations ranging from 80 to 195 min/week, which fell short of the recommended 150–250 min/week. Consequently, the discrepancies between their findings and ours can likely be attributed to the suboptimal duration and dosage of the interventions included in their review (Harris et al., 2015). In contrast, the majority of studies included in our meta-analysis adhered to the recommended physical activity guidelines, leading to significant reductions in both BMI and body fat percentage. Prolonged engagement in mild to moderate-intensity aerobic exercises in a study involving nine participants with intellectual disabilities has been suggested as a viable and effective strategy for reducing subcutaneous fat stores (Merrick et al., 2013). This finding may be age-dependent, as all participants in our study were under 18 years of age, a developmental stage characterized by rapid growth and relatively high energy expenditure compared with adults (Hinckson et al., 2013). However, it is merited to note that many studies included in this review did not report exercise intensity, or how it was modified during the intervention, which limits the accuracy and interpretation of the results. This lack of reporting directly limits the accuracy and interpretation of the results. This ambiguity also strengthens the possibility that variations in exercise intensity explain the divergent outcomes observed in different studies. To optimize the impact of physical activity, it is essential to tailor the intensity of exercise to the individual condition of each participant. This approach is crucial for promoting healthy development, particularly in individuals with IDD.

Our results do not support the efficacy of combined training to yield superior outcomes when compared to MICT alone. However, this finding should be interpreted with significant caution, as it is based on a very small number of studies, and further research is needed to draw a definitive conclusion. The MICT led to significant reductions in BMI and body fat percentage, whereas no discernible impact was observed on WC. The result of this study was inconsistent with Jacinto et al. (2023a) and Jacinto et al. (2023b). A meta-analysis by Jacinto et al. (2023b) indicated that combination training (based on two studies, *n* = 89) was most effective for reducing BMI, while cardiovascular training (based on data from 108 adults with ID) was optimal for reducing WC (Jacinto et al., 2023b). These findings, particularly regarding WC, should be interpreted in the context of their sample, which included a mix of children, adolescents, and adults, with a predominance of adult participants. This population difference is significant, as abdominal obesity (measured by WC) prevalence increases with age (Chen et al., 2019). Therefore, the pronounced effect on WC reported by Jacinto et al. (2023b) may be heavily influenced by the adult data in their meta-analysis and may not be as applicable to a pediatric-only population (Jacinto et al., 2023b). In contrast, our study focused on children and adolescents, who are in a rapid growth phase and possess higher metabolic rates compared to adults (Pontzer et al., 2021), making the effect of exercise on abdominal obesity unclear.

**Figure 7:**
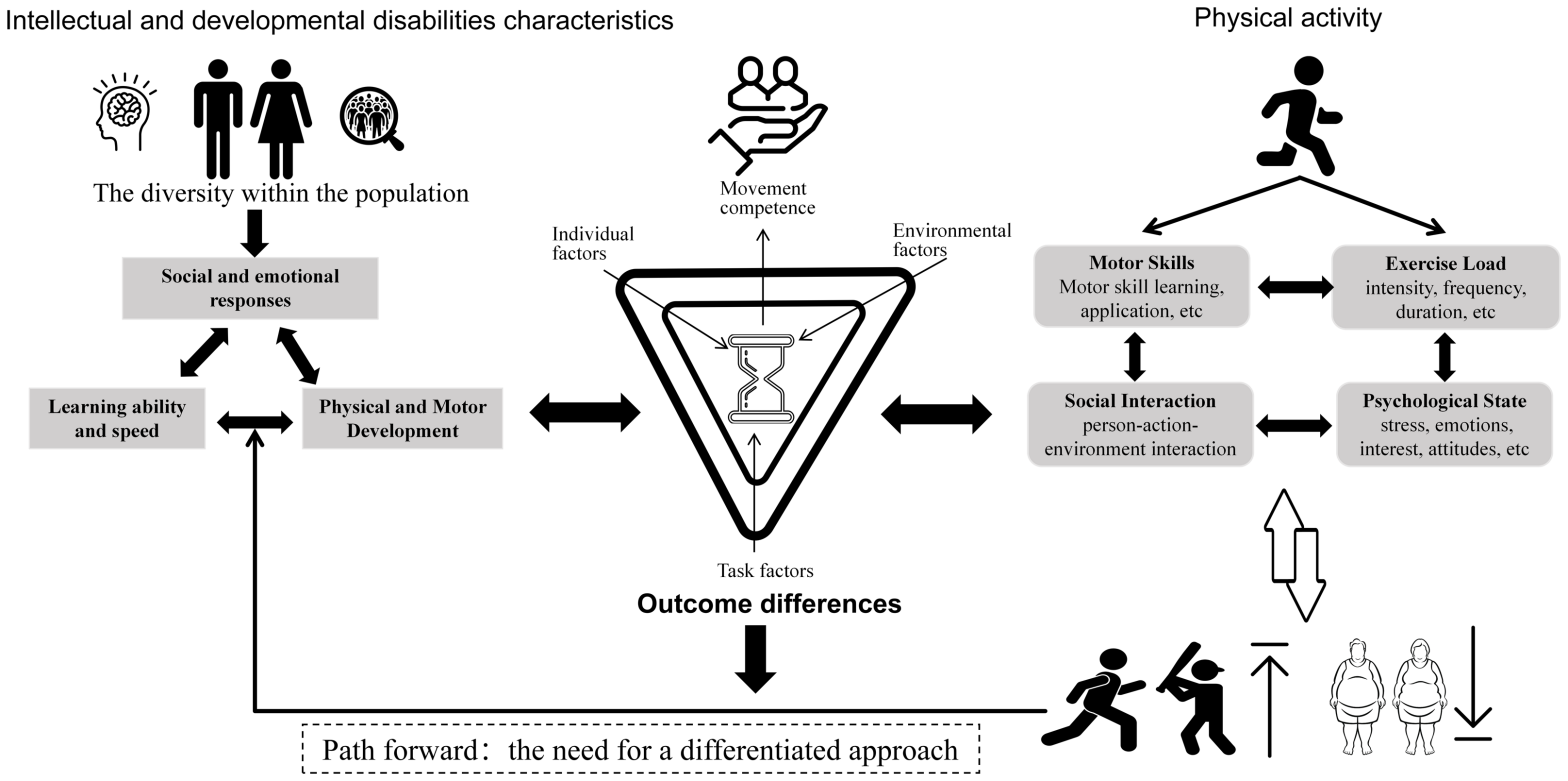
Strategies for physical activity for children and adolescents with IDD.

In summary, our review found that interventions focused on motor skills and MICT are effective strategies for improving FMS and body composition in this population. In contrast, other approaches, such as virtual reality (VR) games or combined training, currently have limited empirical support based on our assessment of the evidence quality. These inconsistent results may be attributed to significant individual heterogeneity, which complicates the control of variables during interventions. Additionally, such interventions might be difficult for individuals with intellectual disabilities to comprehend or access (Naaldenberg et al., 2013). This underscores the need for higher-quality RCTs and a differentiated approach to confirm these findings and enhance confidence in the direction and magnitude of the observed effects (Fig. 7).

## Limitations

This study has several limitations. First, our review was restricted to studies published in English, which may have introduced a language bias by excluding relevant research. Additionally, many studies failed to detail how intensity was set, monitored, or modified, thereby limiting the accuracy of our interpretation and precluding dose–response analysis. Moreover, our finding regarding combined training should be interpreted with caution, as it was based on a very small number of studies, warranting further research. Finally, the comprehensiveness of our conclusions on body composition is limited, as very few primary studies in our review reported on other important measures such as total energy expenditure or visceral adipose tissue. Furthermore, most studies lacked dietary monitoring. As diet significantly affects body composition, observed changes cannot be attributed solely to physical activity.

## Conclusion

This systematic review demonstrated a significant impact of physical activity on FMS and body composition in children and adolescents with IDD, while illuminating broader implications for developmental health. The robust improvements in locomotor and object control skills highlight that movement competence is not merely a physical outcome but a critical enabler of holistic development, fostering cognitive engagement, social interaction, and lifelong physical activity patterns. These results have direct practical applications for clinicians, adapted physical education (APE) specialists, and therapists.

##  Supplemental Information

10.7717/peerj.20946/supp-1 Supplemental Information 1 Search Strategy

10.7717/peerj.20946/supp-2 Supplemental Information 2 PRISMA 2020 checklist

10.7717/peerj.20946/supp-3 Supplemental Information 3 Reply to the Editor

10.7717/peerj.20946/supp-4 Supplemental Information 4 Meta-analysis R code

10.7717/peerj.20946/supp-5 Supplemental Information 5 Dataset

## Additional Information and Declarations

### Data Availability

The following information was supplied regarding data availability: This is a systematic review/meta-analysis.
