# Supplementary material for: Effects of physical activity on fundamental motor skills and body composition in children and adolescents with intellectual and developmental disabilities: a systematic review and meta-analysis
Source: PeerJ. 2026 Apr 1;14:e20946. doi: 10.7717/peerj.20946 (PMC13050218; doi:10.7717/peerj.20946)
Supplement: Supplemental Information 1 [file peerj-14-20946-s001.docx]

**Additional file 1**

| Database | Search strategy |
| --- | --- |
| Web of Science  （2223） | #1 TS=(exercise) OR TS=(Physical activity) OR TS=(Training) OR TS=(Sports) OR TS=(game) OR TS=(Swim) OR TS=(Run) OR TS=(Walk) OR TS=(Motor Activity) OR TS=(Move)  #2 TS=(Intellectual disability) OR TS=(Mentally retarded) OR TS=(Developmental disability) OR TS=(Intellectual function) OR TS=(intellectual impair) OR TS=(autism) OR TS=(Down syndrome)  #3 TS=(Youth) OR TS=(Adolescents) OR TS=(Teens) OR TS=(Children) OR TS=(Child)  #4 TS=(Basic motor skill) OR TS=(Movement skill) OR TS=(Motor ability) OR TS=(Basic movement skill) OR TS=(Fundamental motor skill) OR TS=(Fundamental movement skill) OR TS=(Gross motor skill) OR TS= (Motor competence) OR TS=(Motor coordination) OR TS=(Motor skill) OR TS=(Motor proficiency) OR TS=(Motor development) OR TS=(Motor performance) OR TS=(Locomotor skill) OR TS=(Object control)  #5 TS=(Obesity) OR TS=(BMI) OR TS=(fat percentage) OR TS=(waist circumference)  #6 TS=(RCT) OR TS=(Randomized controlled trial)  #7 #1 AND #2 AND#3 AND (#4 OR #5) AND #6 |
| PubMed  （1676） | #1 Exercise OR Physical activity OR Training OR Sports OR game OR Swim OR Run OR Walk OR Motor Activity OR Move  #2 Intellectual disability OR Mentally retarded OR Developmental disability OR Intellectual function OR intellectual impair OR autism OR Down syndrome  #3 Youth OR Adolescents OR Teens OR Children OR Child  #4 Basic motor skill OR Move skill OR Motor ability OR Basic movement skill OR Fundamental motor skill OR Fundamental movement skill OR Gross motor skill OR Motor competence OR Motor coordination OR Motor skill OR Motor proficiency OR Motor performance OR Locomotor skill OR Object control  #5 Obesity OR BMI OR fat percentage OR waist circumference  #6 RCT OR Randomized controlled trial  #7 #1 AND #2 AND#3 AND (#4 OR #5) AND #6 |
| Cochrane Library  (547) | (Exercise OR Physical activity OR Training OR Sports OR game OR Swim OR Run OR Walk OR Motor Activity OR Move) AND (Intellectual disability OR Mentally retarded OR Developmental disability OR Intellectual function OR intellectual impair OR autism OR Down syndrome) AND (Basic motor skill OR Movement skills OR Motor ability OR Basic movement skill OR Fundamental motor skills OR Fundamental movement skills OR Gross motor OR Motor competence OR Motor coordination OR Motor skill OR Motor proficiency OR Motor development OR Motor performance OR Locomotor skill OR Object control) AND (Youth OR Adolescents OR Teens OR Children OR Child) AND (RCT OR Randomized controlled trial) AND (Obesity OR BMI OR fat percentage OR waist circumference) |
| Medline（793） | #1 Exercise OR Physical activity OR Training OR Sports OR game OR Swim OR Run OR Walk OR Motor Activity OR Move  #2 Intellectual disability OR Mentally retarded OR Intellectual function OR intellectual impair OR Developmental disability OR autism OR Down syndrome  #3 Youth OR Adolescents OR Teens OR Children OR Child  #4 Basic motor skill OR Movement skill OR Motor ability OR Basic movement skill OR Fundamental motor skill OR Fundamental movement skill OR Gross motor skill OR Motor competence OR Motor coordination OR Motor skill OR Motor proficiency OR Motor development OR Motor performance OR Locomotor skill OR Object control  #5 Obesity OR BMI OR fat percentage OR waist circumference  #6 RCT OR Randomized controlled trial  #7 #1 AND #2 AND#3 AND (#4 OR #5) AND #6 |
