# Supplementary material for: Effects of physical activity on fundamental motor skills and body composition in children and adolescents with intellectual and developmental disabilities: a systematic review and meta-analysis
Source: PeerJ. 2026 Apr 1;14:e20946. doi: 10.7717/peerj.20946 (PMC13050218; doi:10.7717/peerj.20946)
Supplement: Supplemental Information 3 [file peerj-14-20946-s003.docx]

**Response**

Systematic Review Registration

Please explain WHY your PROSPERO registration at <https://www.crd.york.ac.uk/PROSPERO/view/CRD42024509463> listed your start date as 01 January 2024, but your PROSPERO registration was submitted later on 03 February 2024, and state what Review stages had been started and completed before PROSPERO submission.

Response:

Project Timeline

January 1: Project initiation – team begins drafting the detailed study protocol.

January 1 - February 3: Protocol development phase:
• Finalizing methodology
• Designing research tools and data collection templates

February 3: Submission of the completed protocol to PROSPERO for registration (establishing a public record of the research plan).

After February 3: Commencement of evidence synthesis:
• Formal literature searches
• Study screening and selection
• Data extraction and analysis

All activities strictly follow the pre-registered protocol.
